# Application of profile fitting method to neutron time-of-flight protein single crystal diffraction data collected at the iBIX

**DOI:** 10.1038/srep36628

**Published:** 2016-12-01

**Authors:** Naomine Yano, Taro Yamada, Takaaki Hosoya, Takashi Ohhara, Ichiro Tanaka, Katsuhiro Kusaka

**Affiliations:** 1 Frontier Research Center for Applied Atomic Sciences, Ibaraki University, 162-1 Shirakata, Tokai, Ibaraki 319-1106, Japan; 2 College of Engineering, Ibaraki University, 4-12-1 Nakanarusawa, Hitachi, Ibaraki, 316-8511, Japan; 3 Neutron Science Section, J-PARC Center, Japan Atomic Energy Agency, 2-4 Shirakata-Shirane, Tokai, Ibaraki 319-1195, Japan

## Abstract

We developed and employed a profile fitting method for the peak integration of neutron time-of-flight diffraction data collected by the IBARAKI Biological Crystal Diffractometer (iBIX) at the Japan Proton Accelerator Research Complex (J-PARC) for protein ribonuclease A and α-thrombin single crystals. In order to determine proper fitting functions, four asymmetric functions were evaluated using strong intensity peaks. A Gaussian convolved with two back-to-back exponentials was selected as the most suitable fitting function, and a profile fitting algorithm for the integration method was developed. The intensity and structure refinement data statistics of the profile fitting method were compared to those of the summation integration method. It was clearly demonstrated that the profile fitting method provides more accurate integrated intensities and model structures than the summation integration method at higher resolution shells. The integration component with the profile fitting method has already been implemented in the iBIX data processing software STARGazer and its user manual has been prepared.

The neutron time-of-flight (TOF) method, which is similar to the X-ray Laue method, uses pulsed neutrons having continuous wavelengths. Because neutron velocities depend on the neutron wavelength, the flight times of the neutrons from their sources (the moderator) through the sample and to the detectors vary. Thus, we can obtain the neutron wavelength by measuring the flight times and separate diffraction peaks at different wavelengths using fixed detectors. In this regard, the TOF method is more efficient than the monochromatic method and can reduce the measurement time^1^. The IBARAKI Biological Crystal Diffractometer (iBIX)^2^ at the Japan Proton Accelerator Research Complex (J-PARC)^3^,^4^, the Protein Crystallography Station (PCS)^5^ at the Los Alamos Neutron Science Center (LANSCE)^6^, and the Macromolecular Neutron Diffractometer (MaNDi)^7^,^8^ at the Spallation Neutron Source (SNS)^9^ are TOF neutron diffractometers for protein single crystals. iBIX, PCS, and MaNDi have installed a H_2_ coupled moderator (CM), partially coupled moderator, and H_2_ decoupled moderator (DM), respectively.

The iBIX, which is installed on beam line BL03 at the Materials and Life Science Experimental Facility (MLF) of J-PARC, is a neutron TOF single-crystal diffractometer used mainly for elucidating the hydrogen, protonation, and hydration structures of biological macromolecules in various life processes. It is equipped with 30 time-resolved scintillator area detectors^10^, each with active areas of 133 × 133 mm (Fig. 1). The flight path lengths from the H_2_ CM to the sample and from the sample to the detectors are 40 m and 490 mm, respectively^2^. At MLF BL03, fast neutrons are generated by the spallation of mercury by 3-GeV proton bombardment^11^, and become thermal neutrons after repeated collisions with hydrogen atoms in the CM. Although most of the moderated neutrons fly toward the sample, some neutrons return to the mercury target. Later, they fly back into the CM and are emitted to the sample. This causes significant broadening of the neutron pulse, leading to an asymmetrically shaped neutron pulse in the direction of the TOF axis^1^. However, the intensities of pulsed neutrons from the CM are stronger than those from DM and poisoned decoupled moderators (PM)^11^.

The peak intensities of neutron diffraction from protein single crystals are relatively weak because neutron beam intensity is lower than X-ray synchrotron intensity, and proteins have lower crystallinity than organic or inorganic compound crystals. Thus, some weak peaks are hidden under the error of strong background generated by strong incoherent scattering of hydrogen atoms in the protein crystals. TOF neutron protein crystallography (NPC) diffraction data processing software has been developed at each diffractometer facility independently. STARGazer^12^, d*TREK modified for wavelength-resolved Laue neutron crystallography^13^, and Mantid^14^ are used at iBIX, PCS, and MaNDi, respectively. Overall, the data quality indices *R*_merge_ and *R*_pim_^15^ of TOF NPC diffraction data are higher than monochromatic synchrotron X-ray and nuclear reactor neutron diffraction data^16^,^17^,^18^,^19^,^20^,^21^,^22^,^23^,^24^. In comparison to monochromatic synchrotron X-ray and nuclear reactor neutron diffraction data, the data processing TOF NPC diffraction data can be improved, and the lowering of *R*_merge_ and *R*_pim_ is a common problem in all neutron TOF single-crystal diffractometers. Therefore, effective methods to determine accurate integrated peak intensities are essential for TOF NPC structural analysis. It has been demonstrated that the application of profile fitting methods to monochromatic X-ray diffraction data can provide more accurate integrated intensities than summation integration methods, especially for weak peaks^25^,^26^,^27^. The profile fitting method for the *x* and *y* detector positions has been implemented in monochromatic X-ray single crystal data processing software such as DENZO^28^, MOSFLM^29^, and XDS^30^. However, it has not yet been demonstrated that this method can be suitably applied to TOF NPC diffraction analysis at iBIX, PCS, and MaNDi.

To the best of our knowledge, one example of applying profile fitting methods in reciprocal space (*Q* space) to the TOF single crystal analysis involved TOPAZ^31^, which is installed on BL 12 at the SNS with a PM. In this example, a Gaussian convolved with two back-to-back exponentials^32^ was used as the fitting function, and was applied to the TOF neutron diffraction data of two single crystals (sapphire and natrolite) and one cocrystal (betaine, imidazole, and picric acid (BIPa)). Because this case did not include protein single crystals measured using a diffractometer with a CM, the same fitting function could not be applied to neutron diffraction data collected from the iBIX. The beam intensity with a CM is more favourable to TOF NPC than a DM or PM. Thus, we attempted to find an appropriate fitting function and develop a profile fitting algorithm for the integration method applicable to the full set of TOF NPC diffraction data collected at the iBIX.

## Results and Discussion

### Evaluation of asymmetric fitting functions

TOF NPC diffraction data collected at iBIX are processed in detector space. Protein diffraction data normally contains more observed reflections than organic or inorganic compounds because of the larger unit cell. Data processing time is an important factor when choosing a profile fitting algorithm. In order to avoid the processing time of transforming from detector space to *Q* space, detector space was adopted in our profile fitting method. First, TOF neutron diffraction data containing the spatial position and TOF for each neutron are converted to histogram data. The number of observed neutrons at *x, y*, and *t* corresponding to the horizontal and vertical detector positions (*x, y*) and a time-of-flight channel (*t*) are recorded. The procedure after this is similar to monochromatic methods and includes peak searching, determination of initial UB matrix, peak indexing, refinement of UB matrix, and peak integration^12^. Because the number of observed neutrons at each pixel of the peak position of three-dimensional (3D) data was insufficient to fit functions accurately, we used projections of the 3D peak in the direction of the TOF axis to improve the statistical precision. Because the pulsed neutron shape was asymmetric when using a CM, the projections of the 3D peaks in the direction of the TOF axis were also asymmetric. Thus, asymmetric functions had to be used in the profile fitting method.

On the other hand, although the procedure is different, the profile fitting method has also been used in TOF neutron powder diffraction studies (Rietveld method^33^), and many asymmetric fitting functions were proposed such as the pseudo-Voigt function convolved with two back-to-back exponentials^34^, and the Ikeda–Carpenter function^35^. A CM is not required to avoid peak overlapping, and fitting functions are utilized not to integrate Bragg intensities but rather to calculate the parameters of the fitting functions using the Rietveld method. The purpose of using fitting functions and the modulator type are different between powder diffraction profile fitting and TOF NPC diffraction profile fitting in iBIX; however, there is a possibility that the proposed fitting functions can be used in both situations.

**Figure 2:**
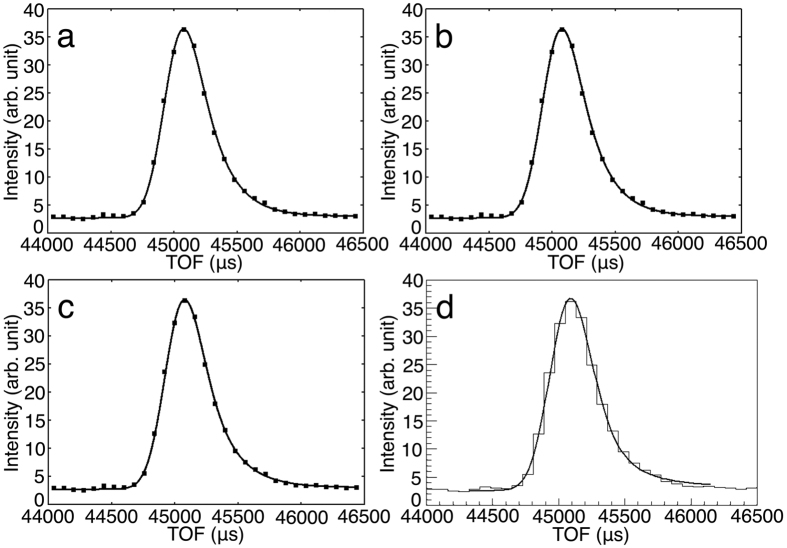
Profile fitting to the *hkl* = −8 1 0 peak from ribonuclease A crystal using four asymmetric functions. (**a**) Gaussian convolved with two back-to-back exponentials fit. (**b**) Pseudo-Voigt function convolved with two back-to-back exponentials fit. (**c**) Gaussian convolved with Ikeda–Carpenter function fit. (**d**) Gaussian convolved with Landau function fit. In panels (**a–c**), SciPy was used to fit the functions and results were plotted by Gnuplot. In panel (**d**), because Gaussian convolved with Landau function contains convolution part in the equation, ROOT was used to fit the function and plot the results. Both four points of the outside regions of the integration region were used as the background region.

In order to determine proper fitting functions for TOF NPC diffraction data, four asymmetric functions were evaluated using strong intensity peaks, with no overlapping, of TOF NPC diffraction data from ribonuclease A collected at the iBIX. These were a Gaussian convolved with two back-to-back exponentials, a pseudo-Voigt function convolved with two back-to-back exponentials, a Gaussian convolved with the Ikeda–Carpenter function derived from TOF profile functions in the GSAS program suite^36^, and a Gaussian convolved with the Landau function^37^. The number of parameters for each fitting function was 5, 6, 6, and 4, respectively. It is important to predict the peak positions and estimate the background accurately to determine the integrated intensity, especially for weak peaks. We adopted a linear function as the background function. Non-linear least square minimization was performed using SciPy or ROOT^38^ software. All four asymmetric functions fit well with strong intensity peaks and significant differences were not found (Fig. 2). It was shown that fitting functions proposed in powder diffraction studies are applicable to TOF NPC diffraction data collected at iBIX. Although the Gaussian convolved with Landau function has the least number of parameters, it is not an analytical function. In order to reduce the processing time and the number of parameters, the Gaussian convolved with two back-to-back exponentials was selected as the most suitable fitting function and used in profile fitting method. This function is analytical and has five parameters: *A, α, β, σ*, and *T*_ph_.

### Implemented fitting function in profile fitting

Further details on the fitting function and its parameters are provided as follows.


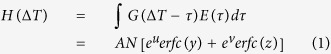


where


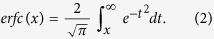


*erfc(x*) is an intrinsic function of the scipy.special package in SciPy.

Here,


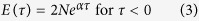


and


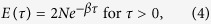


where *α* and *β* are the rise and decay coefficients for the exponentials. *A* is an intensity scale parameter. The original function without *A* is normalized, and the integrated function area is 1. *A* refers to the integrated function area. The normalization factor, *N*, is


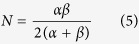


The Gaussian function is


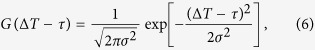


and the Gaussian variance is the coefficient *σ*^2^.

The coefficients *u, v, y*, and *z* are


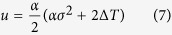



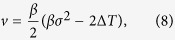



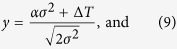



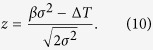


*ΔT* is the difference in TOF between the peak position, *T*_ph_, and the profile point, *T*.


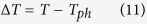


The parameters *α, β*, and *σ* are related to the function profile and are not independent of each other.

### Correlation between fitting function parameters related to profile and TOF

**Figure 3:**
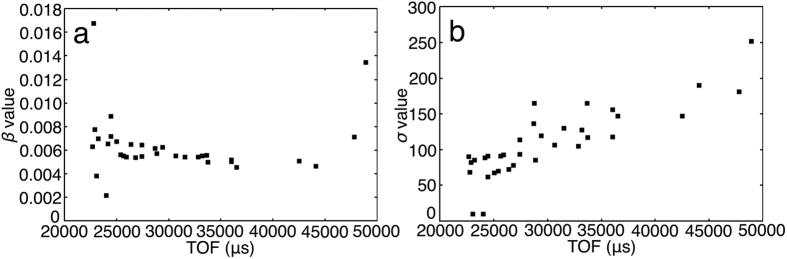
Plots of parameters related to peak profile against TOF in ribonuclease A neutron diffraction data. The peaks whose *I/σ(I*) is over 5 and obtained by one detector located at 51° in 2*θ*_center_ and one crystal orientation were used. (**a**) Plot of parameter *β* against TOF. The peaks whose *β* errors are less than 1 were used. (**b**) Plot of parameter *σ*. The peaks whose *σ* errors are less than 10000 were used.

One of the assumptions in the profile fitting method is that peaks located within a region of reciprocal space or detector space have the same profile^39^. This is the key assumption when determining weak peak profiles. A projection profile in the direction of the TOF axis mainly depends on the TOF, scattering direction, and crystal orientation with respect to the beam. The active area of each detector is 133 × 133 mm and the distance from the sample to the detectors is 490 mm. The maximum scattering angle difference among peaks within a detector is approximately 15°. iBIX contains 30 detectors that each measure different scattering angles. The scattering angle range of the lowest angle detector is from 12.8° to 27.8° and the *Q* range for a wavelength of 4 Å is from 0.35 to 0.75 Å^−1^ where *Q* = 4*π*(sin*θ*)/λ (*θ* is half the scattering angle and λ is the wavelength). Additionally, the scattering angle range of the highest angle detector is from 153.5° to 168.5° and the *Q* range for a wavelength of 4 Å is from 3.06 to 3.12 Å^−1^. Because the maximum scattering angle difference among peaks within a detector is approximately 15°, we assumed that peak profiles at one crystal orientation and one detector change depending only on TOF. In order to prove this assumption, we investigated whether there is a correlation between fitting function parameters related to profile and TOF. The parameters *α, β*, and *σ* are related to the profile. In order to reduce parameter error values, *α* was fixed to a proper value and the *A, β, σ*, and *T*_ph_ parameters were calculated by non-linear least square minimization. Peaks whose *I/σ(I*) were over 5 and with lower parameter errors were selected and the *β* and *σ* parameter values were plotted against TOF at one crystal orientation and one detector. The result from the detector whose 2*θ*_center_ is 51° at one crystal orientation is shown in Fig. 3 as an example. The overall tendency regardless of detectors and crystal orientations is that *β* and *σ* are almost linearly dependent on TOF. Adjacent TOF peaks have similar *β* and *σ* values. Because *β* and *σ* are parameters related to profile shape, these plots show that adjacent TOF peaks have the same profile. Additionally, the maximum scattering angle difference among peaks within a detector is approximately 15°, so adjacent TOF peaks are located within a region of reciprocal space or detector space. We could confirm that the conditions of the profile fitting method are satisfied. The weaker peaks with adjacent TOF have larger parameter errors and the variances of *β* and *σ* in these peaks tend to be larger. In order to determine *β* and *σ* of weak peaks more accurately, adjacent TOF and accurate peaks were selected based on *I/σ(I*), peak intensity, and parameter errors. Accurate peaks with larger TOF occasionally do not have similar *β* and *σ* values to Fig. 3a. Because there are few weak peaks adjacent to those peaks, we do not think it has a negative effect on determination of weak peak profiles.

### Application of profile fitting method to a full set of protein neutron diffraction data

**Figure 4:**
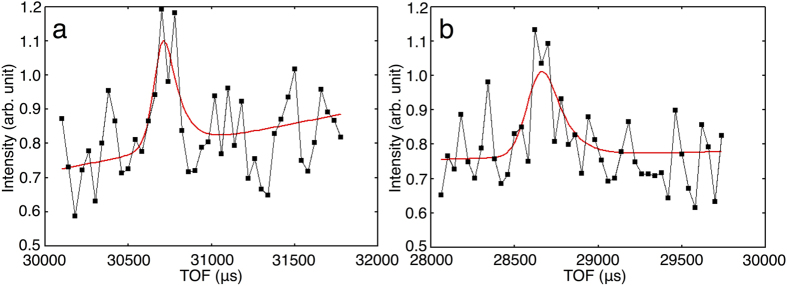
Two examples of profile fitting to the weak peaks from ribonuclease A crystal using Gaussian convolved with two back-to-back exponentials. Red solid line: fitting function. Black points and line: weak peak profile. These peaks were obtained by detector located at 118° in 2*θ*_center_ and one crystal orientation. Both four points of outside regions of the integration region were used as the background region. (**a**) Peak *hkl* is 11 3 16. (**b**) Peak *hkl* is 11 3 20.

We developed a test program and applied it to a full set of ribonuclease A and α-thrombin neutron diffraction data with non-overlapping peaks. The detailed procedure is described in the Methods section. The program could fit asymmetric functions to weak peaks as well as strong ones. The fitting results of two weak peaks obtained by the detector located at 118° in 2*θ*_center_ and one crystal orientation are shown as an example in Fig. 4. Intensity statistics, with peaks *I/σ(I*) larger than 0, were calculated using the iBIX data processing software STARGazer program^12^, and joint refinements of neutron and X-ray data were carried out using the PHENIX program^40^. In order to evaluate the algorithm utility, the intensity and refinement data statistics of the profile fitting method were compared to those of the summation integration method implemented in STARGazer by using the same integration regions, peaks and initial refinement model.

A summary of the intensity data and refinement results of ribonuclease A and α-thrombin is provided in Tables 1 and 2. For both samples, more observed reflections could be used in the profile fitting method than in the summation integration method. This implies that the accuracy of a part of the negative intensity peaks on the summation integration method were improved by the profile fitting method. The higher the resolution, the lower the data quality indices *R*_merge_, *R*_pim_, *R*_work_, and *R*_free_^41^ were on the profile fitting method than on the summation integration method. The greatest improvement in *R*_merge_, *R*_pim_, *R*_work_, and *R*_free_ was observed in the highest resolution shell. The differences in ribonuclease A were 9.5%, 6.8%, 2.3%, and 1.7% and those in α-thrombin were 7.2%, 6.2%, 3.8%, and 4.8%, respectively. Because higher resolution shells contain a higher percentage of weak peaks, it was shown that the profile fitting method is effective with weak peaks, as in previously reported results. *R*_merge_, *R*_pim_, *R*_work_, and *R*_free_ in the overall resolution range were slightly improved over summation integration method. On the other hand, as peak intensities are stronger and background counts are relatively lower in the lower resolution shell, it seems that there is hardly any difference of *R*_merge_, *R*_pim_, *R*_work_, and *R*_free_ between the profile fitting method and summation integration method.

### The reason for the success of the profile fitting method for iBIX data

Two conditions for which the profile fitting method was accurate were proposed^39^. Firstly, peaks located within a region of reciprocal space or detector space have the same profile. Secondly, peak positions can be accurately predicted. It was shown that the first condition was satisfied (See Results and discussion: Correlation between fitting function parameters related to peak profile and TOF) and that weak peak parameters related to peak profile could be determined using more accurate peaks. Recently, detector parameters (distance between each detector and sample, detector position angles, etc.) and 3-axis goniometer offset angles were accurately calibrated and accuracy of peak position prediction was clearly improved. So, the second condition was satisfied. Additionally, an asymmetric fitting function that demonstrated good fitting of peak profiles was determined. In order to fit this function accurately to weak peaks by non-linear least square minimization, proper initial parameters corresponding to each peaks are essential. A procedure to determine proper initial parameter values was introduced (See Methods: Profile fitting procedure of test and implemented programs.) and most weak peaks whose *I/σ(I*) is less than 5 can be fitted using this function.

Because crystallinity of protein single crystals is relatively poor compared to that of inorganic and organic compounds, and iBIX has a CM installed, the projections of the 3D peaks in the direction of the TOF axis are wider and many fitting points can be prepared. It is advantageous for non-linear least square minimization to fit an asymmetric function. Thus, we think that the profile fitting method is more suitable for NPC data analysis obtained by diffractometers with a CM. For these reasons, it is concluded that the profile fitting method for iBIX data has been properly established and that the data statistics of the profile fitting method are an improvement over the summation integration method.

In this study, we definitively demonstrated for the first time that the profile fitting technique is also applicable to comparatively weak peaks and high-background TOF NPC diffraction data using a CM, and that the technique could improve data statistics. It seems that the established profile fitting method is applicable to NPC diffraction data collected from other diffractometers with CMs, and will be able to contribute to developing NPC.

The integration component with the profile fitting method has already been implemented in STARGazer and its user manual has been updated. The software and its manual are available for distribution to iBIX users.

### Future plan

In the future, the accelerator power of J-PARC will be increased to 1000 kW, and we will be able to collect single crystal neutron diffraction data for larger unit cell crystals, including membrane proteins. Because iBIX was designed to measure samples with unit cells up to approximately 135 Å^42^, problems caused by overlapping of adjacent peaks are expected. In order to solve those problems, we are attempting to apply the profile fitting technique to separate overlapped peaks in the TOF direction.

## Methods

### Preparation of ribonuclease A crystal and neutron and X-ray diffraction experiments

Bovine pancreatic ribonuclease A was purchased from Sigma-Aldrich and crystalized as previously described^2^. A crystal with a volume of 6 mm^3^ was soaked in D_2_O solution for 10 days in order to reduce background caused by incoherent scattering from hydrogen atoms. The neutron diffraction data were collected at room temperature over a wavelength range of 1.1 to 5.0 Å. Subsequently, X-ray diffraction data of the same crystal was collected at room temperature in the Photon Factory. The neutron diffraction data were processed using STARGazer^12^ at a resolution of 1.6 Å and X-ray diffraction data were processed using HKL2000^28^ at a resolution of 1.1 Å. Joint refinement of the neutron and X-ray data was performed in PHENIX^40^.

### Preparation of α-thrombin crystals, and subsequent neutron and X-ray diffraction experiments

α-thrombin was purchased from Haematologic Technologies, Inc. (USA) and crystalized as previously described^43^. A crystal with a volume of 3.5 mm^3^ was soaked in D_2_O solution for two weeks in order to reduce background caused by incoherent scattering from hydrogen atoms. The neutron diffraction data were collected at room temperature, using a wavelength range of 2.1 to 6.1 Å. Subsequently, the X-ray diffraction data of the same crystal was collected at room temperature in the Photon Factory. Neutron diffraction data were processed using STARGazer^12^ at a resolution of 2.1 Å and the X-ray diffraction data were processed using HKL2000^28^ at a resolution of 1.6 Å. Joint refinement of the neutron and X-ray data was performed in PHENIX^40^.

### Profile fitting procedure of test and implemented programs

The profile fitting method was performed against peaks observed for every detector and every crystal orientation. A Gaussian convolved with two back-to-back exponentials and a linear function were adopted as fitting and background functions. The regions outside of the integration region in the TOF direction were defined as the background region. At first, the slope and the intercept of the background function were calculated using each peak by non-linear least square minimization. The parameters of the fitting function were calculated using background subtracted intensities by non-linear least square minimization. Proper initial parameter values are essential to obtain accurate results. If the initial parameter values are incorrect, accurate parameters cannot be calculated and incorrect integrated intensities will be obtained. The initial value of *A* is determined from the summation intensity of the projection profile in the direction of the TOF axis. If the obtained values are negative, then a positive value is given instead. Initial *β* and *σ* values are chosen based on previously successful examples. The predicted peak position TOF is used as the initial *T*_ph_ value. The parameters *α, β*, and *σ* are related to the function profile and are not independent of each other. In order to reduce parameter error values, *α* was fixed to a proper value and the *A, β, σ*, and *T*_ph_ parameters were calculated by non-linear least square minimization. The fitting function was integrated and *I/σ(I*) was determined at each peak. The strength of a peak was determined by *I/σ(I*). For example, we can determine that the peaks whose *I/σ(I*) is over 5 are strong and those whose *I/σ(I*) is under 5 are weak. The main assumption in the profile fitting method is that peaks located within a region of reciprocal space or detector space have the same profile^39^. A projection profile in the direction of TOF axis mainly depends on the TOF, scattering direction, and crystal orientation. We assumed that peak profiles at one crystal orientation and one detector change depending only on TOF. In order to obtain more accurate *β* and *σ* values of weak peaks, the *β* and *σ* values of strong and adjacent TOF peaks selected based on *I/σ(I*), peak intensity, and parameter error values were averaged, respectively, and were used as weak peak parameters. *α, β*, and *σ* were fixed, and *A* and *T*_ph_ were calculated by non-linear least square minimization. The fitting functions were integrated and the peak intensities corrected by the Lorentz factor were determined. These procedures were iterated for all detector and crystal orientations.

## Additional Information

**How to cite this article**: Yano, N. *et al*. Application of profile fitting method to neutron time-of-flight protein single crystal diffraction data collected at the iBIX. *Sci. Rep.* **6**, 36628; doi: 10.1038/srep36628 (2016).

**Publisher's note:** Springer Nature remains neutral with regard to jurisdictional claims in published maps and institutional affiliations.

## Figures and Tables

**Figure 1:**
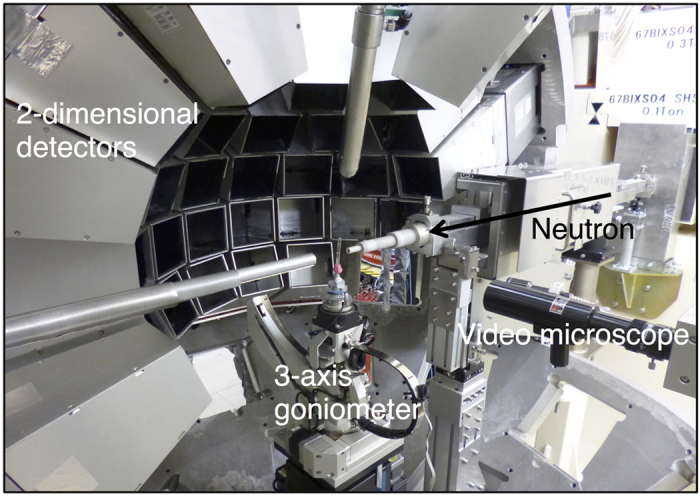
The inside view of iBIX. 30 two-dimensional detectors are installed. The active area of each detector is 133 × 133 mm and the distance from the sample to the detectors is 490 mm.

**Table 1:** Statistics of neutron intensity data and X-ray and neutron joint refinement of ribonuclease A.

Neutron data collection								
Space group	*P*2_1_							
Cell dimensions of *a*(Å)*, b*(Å)*, c*(Å)*, β*(°)^*^				30.29, 38.65, 53.36, 105.30				
Resolution (Å)	Observed reflections		Independent reflections		*R*_merge_		*R*_pim_	
	Profile fitting	Summation integration	Profile fitting	Summation integration	Profile fitting	Summation integration	Profile fitting	Summation integration
15.45–3.44	5,696	5,479	1,525	1,518	0.083	0.081	0.047	0.047
3.44–2.73	7,128	6,888	1,575	1,575	0.083	0.083	0.042	0.043
2.73–2.39	7,825	7,603	1,602	1,600	0.093	0.099	0.046	0.050
2.39–2.17	7,763	7,514	1,567	1,565	0.113	0.118	0.055	0.058
2.17–2.02	7,703	7,461	1,557	1,557	0.139	0.151	0.068	0.074
2.02–1.90	7,409	7,196	1,555	1,555	0.160	0.192	0.081	0.097
1.90–1.80	6,985	6,733	1,577	1,572	0.194	0.244	0.103	0.133
1.80–1.72	6,174	5,931	1,554	1,545	0.206	0.259	0.117	0.150
1.72–1.66	5,496	5,296	1,515	1,508	0.250	0.327	0.151	0.209
1.66–1.60	5,244	5,048	1,492	1,482	0.280	0.375	0.180	0.248
15.45–1.60	67,423	65,149	15,519	15,477	0.114	0.127	0.060	0.068
**X-ray and neutron joint refinement**								
**Resolution (Å)**	**No. of reflections (work/test)**		***R***_**work**_		***R***_**free**_			
	**Profile fitting**	**Summation integration**	**Profile fitting**	**Summation integration**	**Profile fitting**		**Summation integration**	
15.37–2.88	2,478/130	2,472/128	0.180	0.179	0.210		0.211	
2.88–2.29	2,517/133	2,514/133	0.176	0.174	0.222		0.219	
2.29–2.00	2,503/141	2,502/141	0.147	0.152	0.174		0.183	
2.00–1.82	2,465/169	2,462/169	0.157	0.168	0.189		0.210	
1.82–1.69	2,442/119	2,431/118	0.167	0.180	0.209		0.222	
1.69–1.60	2,293/114	2,280/112	0.181	0.204	0.253		0.270	
15.37–1.60	14,698/806	14,661/801	0.170	0.175	0.207		0.213	

^*^Cell constants were calculated from X-ray diffraction data.

**Table 2:** Statistics of neutron intensity data and X-ray and neutron joint refinement of α-thrombin.

Neutron data collection								
Space group	*C*2							
Cell dimensions of *a*(Å)*, b*(Å)*, c*(Å)*, β*(°)^*^				71.72, 72.72, 73.11, 100.51				
Resolution (Å)	Observed reflections		Independent reflections		*R*_merge_		*R*_pim_	
	Profile fitting	Summation integration	Profile fitting	Summation integration	Profile fitting	Summation integration	Profile fitting	Summation integration
20.64–4.51	3,394	3,259	1,738	1,712	0.053	0.053	0.041	0.042
4.51–3.59	4,901	4,661	1,978	1,954	0.058	0.060	0.040	0.042
3.59–3.13	5,391	5,132	2,046	2,023	0.081	0.087	0.054	0.058
3.13–2.85	5,937	5,607	2,033	2,012	0.124	0.137	0.078	0.089
2.85–2.65	5,492	5,122	2,008	1,971	0.154	0.167	0.101	0.112
2.65–2.49	4,464	4,170	1,892	1,851	0.178	0.202	0.125	0.145
2.49–2.36	3,922	3,659	1,814	1,784	0.203	0.234	0.149	0.177
2.36–2.26	3,058	2,904	1,631	1,586	0.226	0.272	0.174	0.215
2.26–2.18	3,072	2,870	1,610	1,557	0.248	0.289	0.192	0.224
2.18–2.10	2,788	2,646	1,514	1,496	0.250	0.322	0.196	0.258
20.64–2.10	42,419	40,033	18,264	17,946	0.103	0.110	0.072	0.079
**X-ray and neutron joint refinement**								
**Resolution (Å)**	**No. of reflections (work/test)**		***R***_**work**_		***R***_**free**_			
	**Profile fitting**	**Summation integration**	**Profile fitting**	**Summation integration**	**Profile fitting**		**Summation integration**	
20.44–3.97	2,435/134	2,398/130	0.149	0.151	0.181		0.192	
3.97–3.15	2,756/130	2,724/132	0.146	0.149	0.186		0.197	
3.15–2.76	2,765/168	2,737/168	0.164	0.169	0.213		0.213	
2.76–2.50	2,619/146	2,557/144	0.160	0.166	0.221		0.221	
2.50–2.33	2,473/130	2,428/130	0.175	0.187	0.225		0.250	
2.33–2.19	2,185/99	2,111/96	0.192	0.216	0.274		0.281	
2.19–2.10	2,105/113	2,071/116	0.208	0.246	0.243		0.291	
20.44–2.10	17,338/920	17,026/916	0.163	0.171	0.209		0.220	

^*^Cell constants were calculated from X-ray diffraction data.
